# Longer Neurophysiological vs. Clinical Recovery Following Sport Concussion

**DOI:** 10.3389/fspor.2021.737712

**Published:** 2021-12-09

**Authors:** Michail Ntikas, Angus M. Hunter, Iain J. Gallagher, Thomas G. Di Virgilio

**Affiliations:** ^1^Department of Psychology, University of Stirling, Stirling, United Kingdom; ^2^Department of Physiology, Exercise and Nutrition Research Group, University of Stirling, Stirling, United Kingdom

**Keywords:** sport-related concussion, intracortical inhibition, Bayesian statistics, transcranial magnetic stimulation (TMS), SCAT5

## Abstract

**Objectives:** The objective of this study was to assess if injury-related alterations in the Sport Concussion Assessment Tool-5 (SCAT5) are matched by changes in transcranial magnetic stimulation-derived intracortical inhibition. We hypothesised that neurophysiological measures would take longer to return to normal than recovery assessed by the SCAT5 following sport related concussion (SRC).

**Methods:** Thirteen male contact sport athletes (20.5 ± 4.5 years), who reported a concussion were recruited from local Rugby and American football clubs. Participants were tested at 4 timepoints throughout the concussion recovery period: within 24 h of concussion (day 0), and at 7, 9, and 11 days after concussion. All participants completed the SCAT5 and underwent TMS to assess cortical silent period duration (CSp), a measure of intracortical inhibition.

**Results:** After concussion CSp significantly declined from day 0 (122 ± 28 ms) to day 11 (106 ± 15 ms) [*F*_(3, 33)_ = 7.80, *p* < 0.001]. SCAT5 measures of symptom number and severity were significantly decreased [symptom number: $\chi_{\left(3\right)}^{2}$ = 30.44, *p* < 0.01; symptom severity: $\chi_{\left(3\right)}^{2}$ = 25.75, *p* < 0.001] between the day 0 timepoint and each of the other timepoints. SCAT5 balance errors (mBESS) decreased significantly [*F*_(3, 33)_ = 19.55, *p* < 0.001] between the day 0 timepoint and each of the other timepoints. CSp and SCAT5 recovery patterns were different. SCAT5 domains recovered faster showing no further significant changes after day 7, whilst CSp was still decreasing between days 7 and 9. Due to the small sample size we also used a Bayesian linear model to investigate the recovery of CSp and mBESS. The posterior distribution of our Bayesian model provided evidence that CSp decreased at day 7 and it continued to decrease at day 9, unlike mBESS which decreased at day 7 and then reached a plateau.

**Conclusion:** There are clinically important discrepancies between clinical and neurophysiological measures of concussion recovery. This finding has important implications for return to play (RTP) protocols and the prevention of complications after sport concussion.

## Introduction

Sport related concussion (SRC) is a public health issue, with almost two-thirds of injuries occurring in children and adults <19 years (Coronado et al., 2015). In the UK, rugby frequently reports SRC with higher prevalence in youth players (Kirkwood et al., 2015; Roberts et al., 2017). However, the true incidence of sport concussion is believed to be higher than reported in most studies due to misdiagnosis and under-reporting (McCrory et al., 2013). The consequences of concussion can be substantial, with neurodegeneration associated with disability, memory impairments and reduced quality of life that likely increase with both severity (Whiteneck et al., 2016) and frequency of concussion (Wilson et al., 2017). Given the scale and severity of these effects, appropriate diagnosis, management, and recovery following concussion is essential.

Despite the potential risks of repeated injury, many concussed athletes choose to either not report their symptoms, or wait until the playing season is over before doing so (Asken et al., 2016). This practise often results from players' reluctance to miss training and games, and pressure from some coaches or parents to play through the injury (Kroshus et al., 2015). To safeguard players against the consequences of concussion the 5th consensus statement on concussion in sport developed comprehensive return to play (RTP) guidelines (McCrory et al., 2017). The statement recommends that following a period of rest, athletes should progress through 5 stages of incremental exercise intensity every 24 h (if symptom free) until they return to normal, uncontrolled play (McCrory et al., 2017). Progression between stages is based on results from the sport concussion assessment tool (SCAT-5), a standardised questionnaire used to evaluate players suspected of having sustained a SRC (Echemendia et al., 2017b). The previous iteration, the SCAT-3, was shown to be limited as an assessment tool 3–5 days post-injury (Echemendia et al., 2017a); the SCAT-5 sought to address these limitations by incorporating additional tests, though it remains unknown if the new iteration improves assessment at days 3–5 post injury. Consequently, combining the SCAT-5 with objective neurophysiological measures may help shed light on the validity of the SCAT5 tool for assessing concussion recovery.

Intracortical inhibition derived from transcranial magnetic stimulation (TMS) reflects inhibitory circuits within the cortico-cortical and corticospinal tract (Kobayashi and Pascual-Leone, 2003). Such inhibitory mechanisms are mediated by the neurotransmitter gamma-aminobutyric acid (GABA) and its type B receptor, GABA_B_ (Inghillerj et al., 1993; Kobayashi and Pascual-Leone, 2003; Scott et al., 2020). Many studies have shown a relationship between concussive injuries and increased inhibitory responses (Chistyakov et al., 2001; De Beaumont et al., 2011; Miller et al., 2014; Scott et al., 2020). Increased GABAergic activity acutely post-injury is thought to be beneficial in helping the brain heal, as rat models show improved sensorimotor and cognitive function associated with a heightened inhibitory response (Demirtas-Tatlidede et al., 2012). However, chronic GABA activation may result in a toxic environment and ultimately hinder brain health (Demirtas-Tatlidede et al., 2012). Furthermore, it is of interest that intracortical inhibition (and electrophysiological parameters in general) appear to outlast cognitive dysfunction and symptom scores, suggesting that physiological recovery are longer than symptom recovery (Livingston et al., 2012; Miller et al., 2014; Pearce et al., 2015). Although intracortical inhibition is thought to be the most consistent TMS parameter in detecting brain alterations following injury (Livingston et al., 2012; Lefebvre et al., 2015; Major et al., 2015; Scott et al., 2020), the intracortical inhibition recovery time course and relationship to diagnostic tools such as SCAT5 remains understudied. Therefore, more studies are needed to identify the altered cortical excitability and inhibition following SRC (Kamins et al., 2017). Hence, the primary aim of this study is to assess if recovery assessed by SCAT5 following SRC is matched by the recovery of intracortical inhibition. We hypothesise that SCAT5 cognitive and motor domains will recover faster than intracortical inhibition.

## Materials and Methods

### Participants and Ethical Approval

A total of 13 male contact sports participants who had suffered from a SRC (7 American football, 6 Rugby) were recruited from local clubs. To be eligible, participants needed to be aged 13–35 y and take part in competitive contact sports, (e.g., Rugby or American Football), at least twice a week. Participants were excluded from the study if they had: (i) a neurological or psychiatric condition (ii) previously suffered from epilepsy, febrile convulsions in infancy, seizures, or had recurrent fainting spells (iii) undergone a neurosurgical procedure (including eye surgery) (iv) any of the following fitted: heart pacemaker, cochlear implant, medication pump, surgical clips, neurostimulator (v) metal in the brain/skull (except titanium). Participants completed a standardised pre-participation questionnaire for underlying health issues (PAR-Q) and TMS suitability (Rossi et al., 2011), as well as providing written informed consent. This study was carried out following ethical approval from the local ethics committee and is in keeping with the latest iteration of the Declaration of Helsinki.

### Study Design

**Figure 1:**
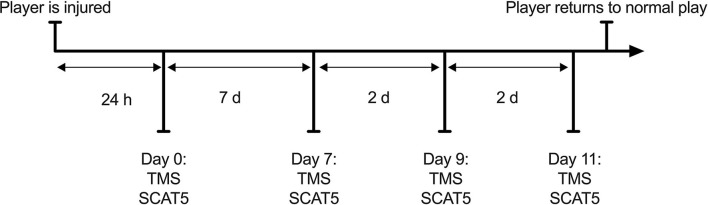
Study flowchart. Participants followed the RTP protocol as advised by their team medic and attended the experiment sessions based on the stage of recovery they were on.

Players with a suspected concussion were encouraged to take part in the study by coaching staff. Concussions were not diagnosed by a physician, players were removed from the pitch if any visible signs of concussion (e.g., dazed look, confusion, balance impairments) were observed, according to guidance set out by Sport Scotland (2018). This approach reflects what happens in an amateur context, as most teams at this level do not have access to team physicians to diagnose any injury. The first stage of the graduate RTP protocol requires 7 days complete rest, following which players can progress through stages 2–6 every 24 h if no symptoms appear. We believe that participants in our study only experienced mild concussions as they were able to progress between stages without additional delays. Participants were tested at 4 time points throughout their recovery, within 24 h (18 h ± 4) of injury (day 0) and then at days 7, 9, and 11 post-injury, corresponding to stages 2, 4, and 6 of their graduated return to play following the Scottish Sport Concussion Guidelines (Sport Scotland, 2018; Figure 1). Participants were asked to refrain from exercise, caffeine and alcohol use 48 h before the sessions. Participants underwent the same protocol during each testing session, completing the SCAT5 (neurological function) and TMS (corticomotor inhibition) evaluation (Figure 1). Data were collected by a single individual trained by members of a research team with >20 years experience in neuromuscular assessments (Di Virgilio et al., 2019).

### Sporting Concussion Assessment Tool 5th Edition

The SCAT5 is comprised of multiple domains: symptom evaluation, cognitive and neurological screening, and the modified balance error scoring system (mBESS). Symptom evaluation requires the participant to rate their well-being on 22 different questions at the time of testing. Each question scales from 0 (non-existent) to 6 (severe), giving a maximum possible score of 132, with a higher overall score indicating more symptoms and increased severity. The cognitive screening section is made up of the standard assessment of concussion (SAC), which tests orientation, immediate memory, concentration, and delayed recall. The neurological assessment covers multiple domains (see Supplementary Material 1) giving either yes or no responses to assess for any abnormal conditions. The mBESS requires participants to hold 3 stances (on two feet, one foot and in tandem stance) for 20 s with eyes closed. A score is obtained for each stance by adding 1 point for each error made during the 20 s. An error was recorded when the athletes moved their hands from the iliac crest, opened their eyes, made a step, had a fall, displayed hip abduction or flexion beyond 30°, lifted their forefoot or heel from the testing surface or remained out of the proper testing position for more than 5 s. The mBESS can be subjective in nature, with some of its tasks showing low interrater and intrarater variability (Finnoff et al., 2009). For the purposes of our study all errors were recorded by the same individual at all times and only the tasks found to have an acceptable intrarater variability were used.

### Electromyography

All assessments took place indoors. Participants were seated in either a customised load cell, if assessed at the club training ground, or isokinetic dynamometer (Biodex System 4, New York, NY, United States), if assessed in our neurophysiology laboratory, with their knee at 60° flexion (full leg extension 0°). Participants undertook a standardised warm-up of 3 × 50% then 3 × 70% of their perceived maximum voluntary contraction (MVC) with 30 s recovery between exertions. Participants then performed 3 MVCs of 5 second duration each, from which 20% of MVC was calculated for the TMS. Electromyography (EMG) was used to measure surface amplitude of the rectus femoris muscle. Pilot testing and previous work within our laboratory (Di Virgilio et al., 2016, 2019) has shown that the rectus femoris is the best muscle in the quadriceps femoris for the elicitation and recording of inhibitory/excitatory responses. Responses were recorded using a wireless system (Biopac Systems, Inc. Goleta, CA, USA) and disposable Ag/AgCl surface electrodes (Vermed, Devon, UK). Following the surface EMG for non-invasive assessment of muscles (SENIAM) recommendations (Hermens et al., 2000), electrode positions were selected, shaved, and cleaned with an alcohol wipe. Electrodes were then positioned 2 cm apart. Data were sampled at 2 kHz and filtered using 500 Hz low and 1.0 Hz high band filters.

### Transcranial Magnetic Stimulation

**Figure 2:**
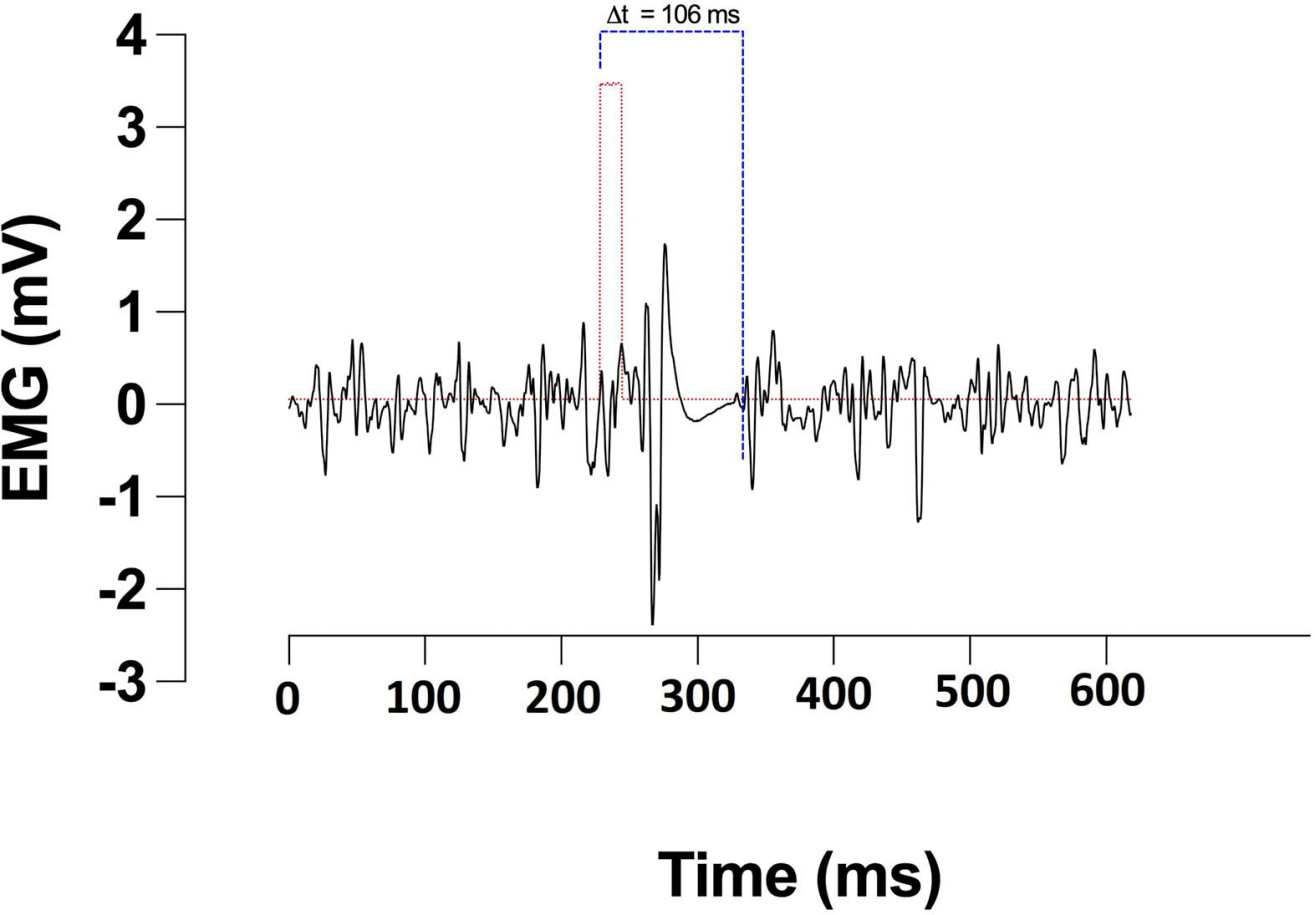
CSp duration.

Corticomotor inhibition was assessed in the rectus femoris of participants' self-reported dominant leg by applying TMS contra-laterally over the primary motor cortex (M1). Single pulse magnetic stimulations of 1 ms duration were applied using a magnetic stimulator and 110 mm double cone coil (Magstim 2002 unit, The Magstim Company Ltd., Whitland, UK). Participants were instructed to extend their leg at ~20% of their MVC guided by visual feedback of force production. Optimal coil position for generating motor evoked potentials (MEPs) in the rectus femoris was identified by placing the coil lateral to the vertex and moving by 1 cm steps. The coil position producing the largest MEP was marked with indelible ink and used for the rest of the study (Goodall et al., 2009). Active motor threshold (aMT) was identified through participants holding 20% of their MVC whilst single pulse stimulations were applied. Intensity started at 20% of the stimulator's maximum output, increasing by 5% increments if four out of five stimulations did not elicit a visible MEP (Wilson et al., 1995). To assess corticomotor inhibition, participants were instructed to perform a 5s MVC during which a stimulation of 130% aMT was applied. Participants were instructed to maintain the contraction through the stimulation and briefly after. This was repeated 3 times with a rest period of 30 s between contractions. The cortical silent period (CSp) duration was defined as the time from the stimulus artefact to the resumption of normal voluntary EMG activity and was manually assessed (Figure 2).

### Data Analysis Plan

CSp, SCAT-5 and balance data were analysed in IBM SPSS v27. Repeated measures ANOVAs were used to investigate the pattern of recovery after a SRC with Time (days) being the within variable; *post-hoc* comparisons were corrected with the Bonferroni method. In the case of non-normally distributed data, non-parametric alternative tests were used (Friedmann tests with Wilcoxon signed-rank as *post-hoc*—please refer to the Supplementary Material for details of normality checks). Statistical significance was set at *p* < 0.05.

We also used the R package RStanarm (Goodrich et al., 2020) to carry out the Bayesian alternative to the frequentist analyses described above and used the posterior distributions to examine the probabilities of the presence of an effect (Swinton et al., 2018). Small sample sizes are commonplace in concussion research, with the majority of existing TMS studies reporting data from a limited number of athletes (Miller et al., 2014; Pearce et al., 2015; Edwards and Christie, 2017). Bayesian analyses provide direct probabilistic comparisons between groups/treatments and are suited to sport science studies as they provide more defence against over-confidence in small sample sizes (Mengersen et al., 2016; Borg et al., 2018) compared to frequentist techniques. The RStanarm package provides an interface to the Stan probabilistic programming language, which uses Hamiltonian Markov Chain Monte Carlo (MCMC) method to generate MCMC chains used to characterise the posterior distribution (Hoffman and Gelman, 2014). After defining the model and priors, the other values set in the call to RStanarm were 4 MCMC chains with 2,000 iterations each and a burn-in period of 200 iterations. The priors used can be found in the Supplementary Material 1. Briefly the prior for the CSp was a normal distribution centred on the average CSp found in healthy participants in our laboratory and a standard deviation 2.5 times the standard deviation from healthy participants. Backward difference coding (found in Supplementary Material 1) was used to compare the mean of the dependent variable (CSp) for one level of the independent variable (Time in days) to the mean of the dependent variables for the prior adjacent level; day 7 compared to day 0, day 9 compared to day 7; day 11 compared to day 9.

## Results

### Participants/Outliers

Thirteen athletes completed the study. All athletes were male with a mean age of 20.5 (±4.5) years and were playing for local amateur rugby and American football clubs (Table 1). One of the 13 concussed participants was identified as an outlier and excluded from the analyses because the CSp values immediately after concussion were physiologically inconsistent (abnormally high) and more than 3 IQRs from the median of the sample (Supplementary Material 1).

**Table 1:** Participants' characteristics; median (IQR).

**Characteristic**	**Value**
*N*	13
Age (y)	20 (6.5)
Number of previous concussions	1 (2)
Time since last concussion (months)	13 (13)

*N, number of participants*.

### Recovery Patterns

The CSp of the concussed group significantly declined from day 0 to day 11 [*F*_(3, 33)_ = 7.80, *p* < 0.001]. Specifically, inhibition decreased between the day 0 and day 9 (*p* < 0.001) and day 11 (*p* = 0.002) post-concussion time points (Table 2).

**Table 2:** Descriptive statistics for symptom number and severity, mBESS and CSp.

	**Day 0**	**Day 7**	**Day 9**	**Day 11**
Symptoms number	8 (4.25)	2.5 (4.75)^*^	0 (1.25)^†^	0 (0.5)^†^
Symptoms severity	11.5 (14.5)	2.5 (6)^*^	0 (1.25)^†^	0 (0.5)^†^
mBESS	10.92 (3.26)	4.67 (4.7)^*^^¥^	4.17 (3.33)^†^	3.42 (3.09)^†^
CSp (seconds)	0.116 (0.015)	0.110 (0.017) ^¥^	0.106 (0.015)^*^^¥^	0.106 (0.014)^*^

*Median (IQR) for Number of Symptoms and Severity of symptoms, Mean (SD) for mBESS and CSp.*

* *denotes significant difference from Day 0;*

† *denotes significant difference from Day 0 and no difference from Day 7.*

¥ *denotes evidence of difference (90% HDI) from the previous timepoint in the Bayesian model*.

The number of concussion symptoms significantly decreased [$\chi_{\left(3\right)}^{2}$ = 30.44, *p* < 0.01] between the immediate (day 0) and day 7 (*p* = 0.002), 9 (*p* = 0.002) and day 11 (*p* = 0.002) timepoints post-concussion (Table 2); when corrected for multiple comparisons with the Bonferroni method the differences remained significant. Similarly, symptom severity significantly decreased [$\chi_{\left(3\right)}^{2}$ = 25.75, *p* < 0.001] between day 0 and day 7 (*p* = 0.003), day 9 (*p* = 0.002), and day 11 (*p* = 0.003) post-concussion (Table 2); the uncorrected comparison between days 7 and 9 was significant (*p* = 0.011) but did not remain so after Bonferroni correction. The cognitive tasks of SCAT5 failed to reach levels of statistical significance (*p* > 0.05; see Supplementary Material 1). Lastly the number of errors on the mBESS decreased significantly [*F*_(3, 33)_ = 19.55, *p* < 0.001] between the day 0 and days 7 (*p* < 0.001), 9 (*p* < 0.001) and 11 (*p* < 0.001) post-concussion; all other comparisons were non-significant.

**Figure 3:**
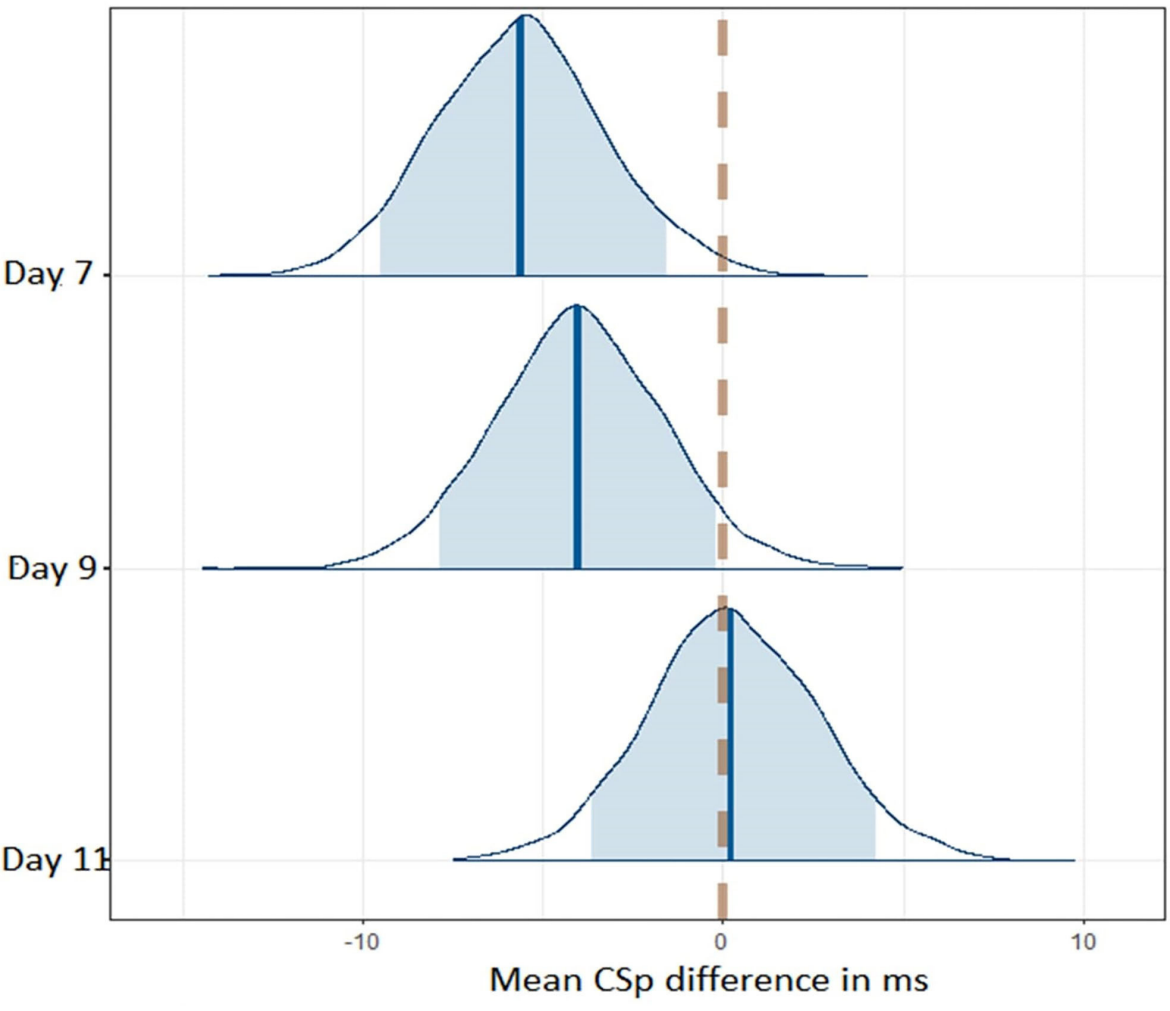
Posterior distributions for CSp differences between successive timepoints across the time course of the study. The dashed line represents no difference in CSp between successive timepoints. The shaded area represents the 90% higher density interval (HD).

**Figure 4:**
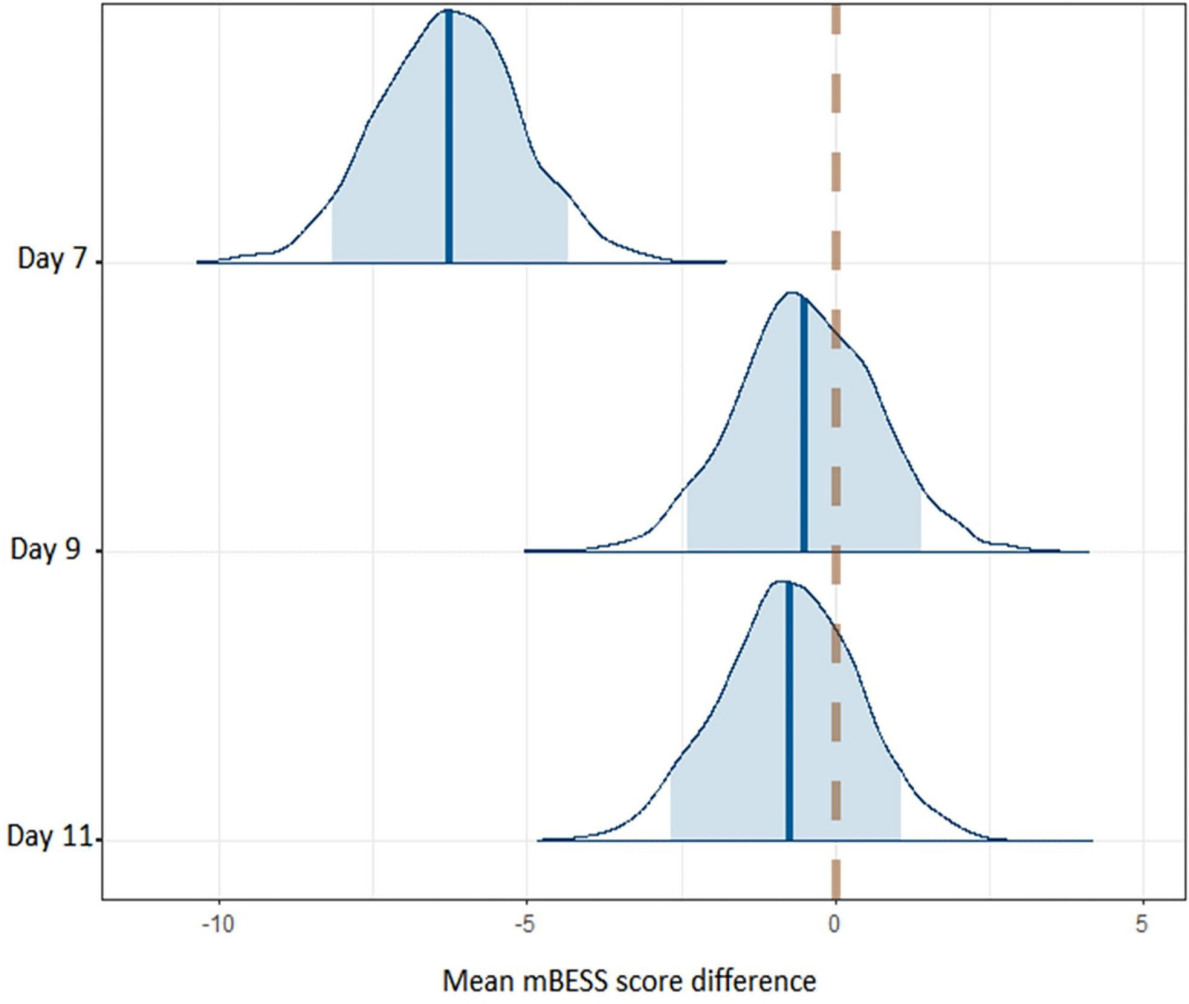
Posterior distributions for balance error differences between successive timepoints across the time course of the study. The dashed line represents no difference in balance errors between successive timepoints. The shaded area represents the 90% higher density interval (HDI).

We used Bayesian methods to generate posterior distributions so we could assess a proportion of response (Swinton et al., 2018) for CSp and mBESS over the recovery period. For CSp values the posterior distributions with a 90% highest density interval (HDI) provide evidence that CSp duration is decreased at day 7 compared to day 0 after concussion and from day 7 to day 9 post-concussion (Figure 3). More than 90% of each of these posterior distributions lies below a difference of zero (Figure 3). The posterior distribution for the difference in CSp duration between days 9 and 11 is centred on zero suggesting no difference in CSp between these timepoints (Figure 3). Concerning the mBESS in SCAT5, the posterior distributions with a 90% HDI provide evidence that the number of errors is decreased at day 7 compared to immediately after concussion (Figure 4). The posteriors distributions provide evidence that the number of balance errors remains similar between the days 7, 9 and 11 post-concussion (Figure 4).

## Discussion

The aim of this study was to examine if the functional recovery assessed by SCAT5 following a SRC is matched by the recovery of neurophysiological parameters. The combination of an objective measure of neurophysiological changes (TMS) with subjective clinical measures (SCAT5) provided new insights into recovery after SRC; indeed, our findings show a decrease in clinical and neurophysiological measures assessing concussion 9 and 11 days post injury, compared to immediately after the SRC. The differing recovery rates between SCAT5 domains and CSp corroborate our hypothesis and further suggest that neurophysiological measures are still altered in the absence of clinical symptoms after SRC.

**Figure 5:**
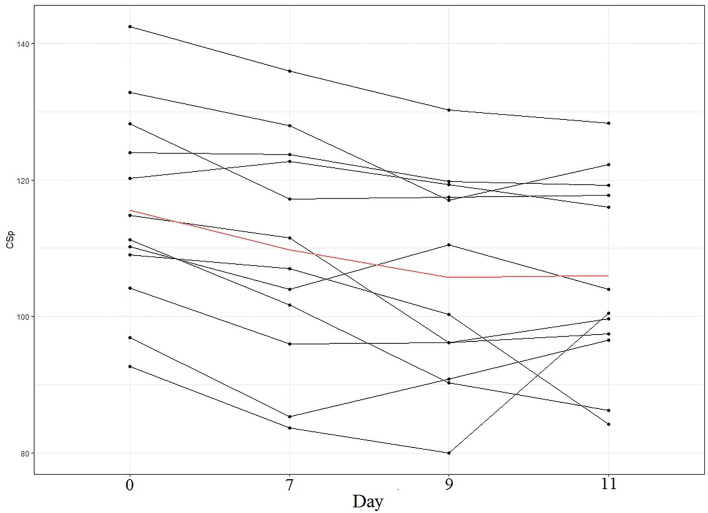
CSp (in ms) pattern per participant. Each line represents a participant in our sample. The red line represents the mean of our sample.

Although both CSp and SCAT5 measures (number and severity of symptoms, and balance errors) were decreased in the week after SRC, their recovery followed a different pattern. The SCAT5 domains that were elevated after a concussion at day 0 reached a plateau at the 7th day post-concussion, showing no significant differences from day 7 to day 11 postconcussion. Furthermore, symptom number and severity at days 7-11 are comparable to normative values from a similar, healthy population (Tucker et al., 2020) suggesting that these domains had returned to pre-injury levels by the time participants had completed to RTP protocol. Conversely, CSp was still decreasing at day 7 and day 9 post-concussion, suggesting a slower recovery. This pattern of decrease was present in the majority of athletes (Figure 5). In the context of SRC recovery it is also interesting that postural control measured by the mBESS appears to recover sooner than intracortical inhibition. Both measures are indices of motor control (Kobayashi and Pascual-Leone, 2003; Clark et al., 2010), and the differing recovery trajectory suggests that gross measures (e.g., postural control/balance) may not be reliable in detecting post-acute concussive impairments of the motor system. Furthermore, relying solely on the SCAT5 as a diagnostic tool could mean that players might be deemed fit for RTP whilst the motor system is not fully recovered. This notion is supported by previous studies reporting increased musculoskeletal injury risk in previously concussed players (Nordström et al., 2014; Lynall et al., 2015; Cross et al., 2016), suggesting a persistent motor dysfunction even when clinical symptoms have fully resolved. Our findings also support the argument that although SCAT5 is considered useful immediately post-injury for the diagnosis of concussion, its utility decreases few days after the SRC, making its use for RTP assessment questionable (Echemendia et al., 2017b).

Research on cortical inhibition in concussion management has yielded mixed findings so far; the reported altered CSp in the acute post-concussion phase has been shown to either resolve after a fortnight (Pearce et al., 2015), or remain increased even 2 months after injury compared to controls (Miller et al., 2014). Interestingly, one study revealed a pattern of recovery in which CSp decreases 1 and 2 weeks after the initial concussion, only to increase again 1 and 2 months later (Edwards and Christie, 2017). Our study provides further evidence for this decrease of inhibitory response after a period of rest (9–11 days in our case) following a SRC, compared to immediately after the SRC. This decrease in inhibitory response, followed by a period of stability, is an indicator that the physiological alterations caused by the SRC have been resolved 9 days post-concussion. Due to the lack of a baseline measure we cannot be certain that CSp returns to normal pre-injury levels, however, the observed plateau 9–11 days post-injury indicates a stabilisation of CSp. This CSp alteration after a SRC can have both short-term and long-term implications. CSp length is mediated by the GABA_b_ neurotransmitter (Kobayashi and Pascual-Leone, 2003) and although, a short term GABA increase might be part of a protector mechanism for the brain, increased GABA has been linked with hindering of learning processes such as verbal and motor learning, with decreased GABA being necessary for motor learning acquisition (Mondadori et al., 1996; Stagg et al., 2011). In the long-term, GABA increase can have detrimental effects to brain's health and development. To better understand the relationship between brain-muscle pathway integrity and SRC, researchers using TMS in a concussion-related context should endeavour to formalise standard protocols to ensure data consistency across different studies.

### Limitations

One major limitation of this study is the lack of a pre-concussion baseline measure. Athletes playing in local American football or rugby teams were asked to participate to the study upon suffering an SRC and having a baseline measure for all local athletes was not a feasible option. Further, from a methodological standpoint future studies would benefit from assessing inhibitory responses from whole muscle groups as opposed to single muscles, in order to understand the overall effects of SRC on the motor system. Another limitation of our study is the relatively small sample size; however, as the sample size in this case could not have been predetermined (unclear how many athletes will suffer a SRC) we believe that Bayesian statistics provide an elegant solution to address this problem, allowing us to draw inferences from smaller amounts of data (Mengersen et al., 2016; Borg et al., 2018). Moreover, the findings of this study should be interpreted with caution since the athletes assessed belonged to a specific demographic [20.5(±4.5) y.o., Caucasian males].

## Conclusion

In this study we showed that neurophysiological parameters and clinical symptoms following SRC recover with different time courses. By using a Bayesian method to analyse the data we were able to gain more information about SRC recovery from a small sample of athletes, providing further support for the importance of using alternative statistical methods in the field of sport science. In particular, motor function measured through postural control recovered sooner than intracortical inhibition, suggesting that although concussed players may appear healthy and fit to RTP, they may still have underlying and subtle motor impairments. Our findings suggest that using solely clinical measures to assess whether players are fit to resume play may put them at risk of either sustaining another injury or develop other complications in later life. The relationship between clinical and neurophysiological measures of SRC recovery should be investigated further with studies including a pre-concussion baseline, larger sample sizes and more post-concussion timepoints.

## Data Availability Statement

The raw data supporting the conclusions of this article will be made available by the authors, without undue reservation.

## Ethics Statement

The studies involving human participants were reviewed and approved by NHS, Invasive or Clinical Research (NICR) Committee. Written informed consent to participate in this study was provided by the participants' legal guardian/next of kin.

## Author Contributions

TD and AH designed the experiment and performed the data collection. MN and IG analysed the data. MN and TD drafted the manuscript. MN, TD, IG, and AH reviewed and approved the manuscript. All authors contributed to the article and approved the submitted version.

## Conflict of Interest

The authors declare that the research was conducted in the absence of any commercial or financial relationships that could be construed as a potential conflict of interest.

## Publisher's Note

All claims expressed in this article are solely those of the authors and do not necessarily represent those of their affiliated organizations, or those of the publisher, the editors and the reviewers. Any product that may be evaluated in this article, or claim that may be made by its manufacturer, is not guaranteed or endorsed by the publisher.
