# Supplementary material for: Longer Neurophysiological vs. Clinical Recovery Following Sport Concussion
Source: Front Sports Act Living. 2021 Dec 9;3:737712. doi: 10.3389/fspor.2021.737712 (PMC8695881; doi:10.3389/fspor.2021.737712)
Supplement: Supplementary file 1 [file Data_Sheet_1.pdf]

SUPPLEMENTARY MATERIAL 1

1. **SCAT5 link:** https://bjsm.bmj.com/content/bjsports/early/2017/04/26/bjsports-2017-097506SCAT5.full.pdf

2. **Bayesian analysis priors for CSp:**

Normal distribution with a Mean= 120.3 and SD= 21.9; specific information about the rest of the priors can be found in the script below.

3. **R Script for backward difference coding:**

```
rm(list=ls())

library(readr)

library(dplyr)

library(tidyr)

library(ggplot2)

theme_set(theme_bw())

# load rstanarm & useful libs

library(rstanarm)

library(bayesplot)

library(tidybayes) # working with 'tidy' bayesian models

library(haven)

library(emmeans)


# For CSp:

back_contrast_mat <- matrix(c(-3/4, rep(1/4,3),  rep(-1/2, 2), rep(1/2, 2),  rep(-1/4, 3), 3/4), ncol = 3)

back_contrast_mat

# mutate Time to factor

CSp_repeated_measures_long <- CSp_repeated_measures_long %>% mutate(Time.f = as.factor(Time))

# assign the backward difference coding to the time factor (Time.f)

contrasts(CSp_repeated_measures_long$Time.f) <- back_contrast_mat
```

```
# create the model

rep_meas <- stan_lmer(CSp ~ Time.f + (1 | Subj), data = CSp_repeated_measures_long,
                 prior_intercept = normal(location = mean),
sd(CSp_repeated_measures_long$CSp), autoscale = FALSE),
                 prior = normal(location = 0, scale = 10, autoscale = FALSE),
                 prior_aux = exponential(rate = 1, autoscale = TRUE),
                 prior_covariance = decov(regularization = 1, concentration = 1, shape = 1, scale
= 1),
                 seed = 123)

# we can ignore the random intercepts, just there to account for non-independence; summary
with 90% HDI

summary(rep_meas, pars = c("(Intercept)", "sigma"), regex_pars = c("Time", "Sigma"), prob
= c(0.05, 0.5, 0.95))

prior_summary(rep_meas)

plot(rep_meas, regex_pars = ("Time"), prob = 0.9) + geom_vline(xintercept = 0, colour =
"chocolate4")

plot(rep_meas, plotfun = 'mcmc_areas', regex_pars = ("Time"), prob = 0.9) +
geom_vline(xintercept = 0, colour = "chocolate4", alpha = 0.5, size = 2, linetype = 'dashed')


# For Balance:

back_contrast_mat <- matrix(c(-3/4, rep(1/4,3),  rep(-1/2, 2), rep(1/2, 2),  rep(-1/4, 3), 3/4), ncol = 3)

back_contrast_mat

# mutate Time to factor

Scat_BESS_long <- Scat_BESS_long %>% mutate(Time.f = as.factor(Time))

# assign the backward difference coding to the time factor (Time.f)

contrasts(Scat_BESS_long$Time.f) <- back_contrast_mat

# adjusted location of the intercept prior to the mean of all the data

# adjusted scale of the intercept prior to the sd of all the data

rep_meas <- stan_lmer(BESS ~ Time.f + (1 | Subj), data = Scat_BESS_long,
             prior_intercept = normal(location = mean(Scat_BESS_long$BESS), scale =
sd(Scat_BESS_long$BESS), autoscale = FALSE),
             prior = normal(location = 0, scale = 10, autoscale = FALSE),
             prior_aux = exponential(rate = 1, autoscale = TRUE),
```

prior_covariance = decov(regularization = 1, concentration = 1, shape = 1, scale = 1),

seed = 123)

summary(rep_meas, pars = c("(Intercept)", "sigma"), regex_pars = c("Time", "Sigma"), prob = c(0.05, 0.5, 0.95))

prior_summary(rep_meas)

Note plots below are the posteriors for the DIFFERENCES not the actual mBESS!!

plot(rep_meas, regex_pars = ("Time"), prob = 0.9) + geom_vline(xintercept = 0, colour = "chocolate4")

plot(rep_meas, plotfun = 'mcmc_areas', regex_pars = ("Time"), prob = 0.9) + geom_vline(xintercept = 0, colour = "chocolate4", alpha = 0.5, size = 2, linetype = 'dashed')

4. Outliers

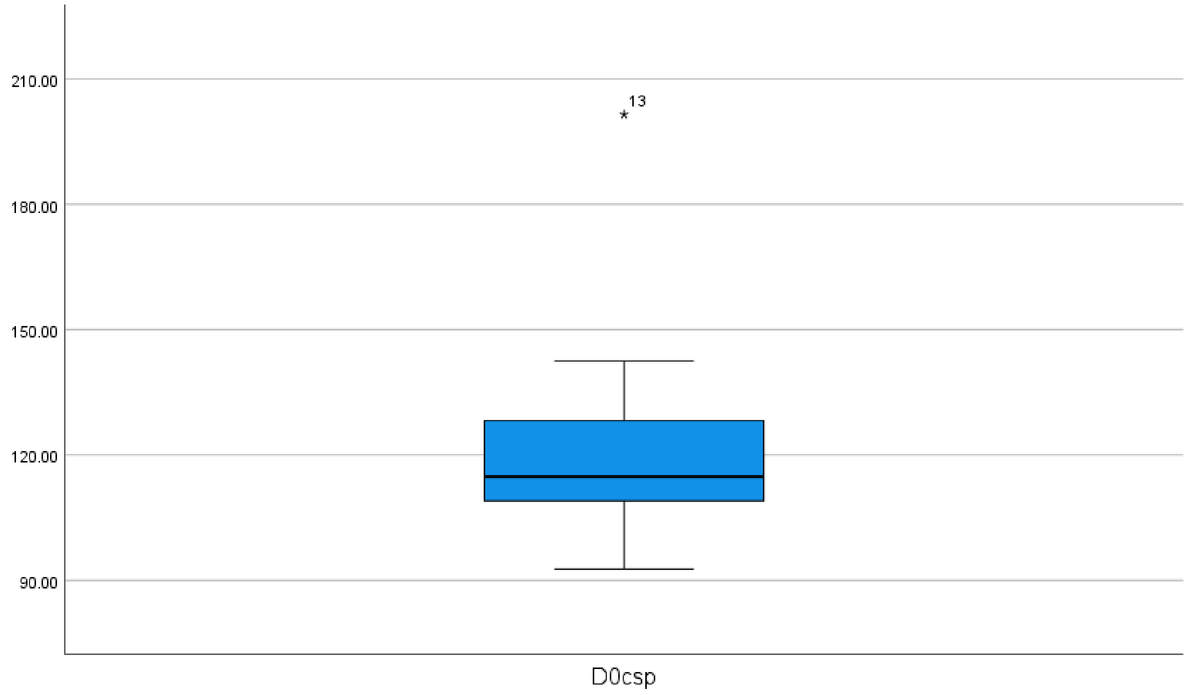

5. Normality checks

Descriptive Statistics & normality tests for CSp and mBESS

	D0 CSp	D7 CSp	D9 CSp	D11 CSp	D0Balace	D7Balace	D9Balance	D11Balance
Valid	12	12	12	12	12	12	12	12
Mean	115.6	109.7	105.7	106.0	10.9	4.67	4.2	3.4
Std. Deviation	14.7	16.6	15.4	14.4	3.26	4.70	3.33	3.09
Shapiro-Wilk	0.984	0.968	0.945	0.941	0.933	0.885	0.939	0.918
P-value of Shapiro-Wilk	0.994	0.892	0.566	0.505	0.418	0.101	0.487	0.271

Descriptive Statistics & normality tests for SCAT5 Number and Severity of Symptoms

	D0Number	D7Number	D9Number	D11Number	D0Severity	D7Severity	D9Severity	D11Severity
Valid	12	12	12	12	12	12	12	12
Mean	8.83	3.17	0.67	0.83	16.25	4.67	0.67	1.25
Std. Deviation	3.22	3.46	1.07	1.80	10.00	5.99	1.07	2.93
Shapiro-Wilk	0.930	0.858	0.680	0.547	0.859	0.796	0.680	0.511
P-value of Shapiro-Wilk	0.379	0.046*	< .001*	< .001*	0.048*	0.008*	< .001*	< .001*

6. Recovery Patterns; Repeated measures ANOVAs for the cognitive tasks of SCAT

Descriptive statistics Mean(SD):

	Day 0	Day 7	Day 9	Day 11
Immediate recall	21.15 (2.23)	21.23(3.26)	21.77(3.30)	22.92(4.13)
Delayed recall	6.92(1.04)	6.84(1.07)	7.23(1.36)	6.46(1.13)
Concentration	3.62(1.45)	3.85(1.07)	3.92(0.95)	4.15(0.90)

Note. Immediate recall score out of 30; Delayed recall score out of 10; concentration score out of 5.

SCAT5 Immediate recall repeated measures ANOVA:

Within Subjects Effects

Cases	Sum of Squares	df	Mean Square	F	p
Time	26.000	3	8.667	1.102	0.361
Residuals	283.000	36	7.861		

SCAT5 Immediate recall repeated measures ANOVA: (Greenhouse-Geisser correction)

Within Subjects Effects

Cases	Sum of Squares	df	Mean Square	F	p
Time	2.229	1.64	1.36	1.114	0.358
Residuals	22.021	18.0	1.22		

SCAT5 Delayed recall repeated measures ANOVA:

Within Subjects Effects

Cases	Sum of Squares	df	Mean Square	F	p
Time	3.500	3	1.167	1.242	0.310
Residuals	31.000	33	0.939		
